# Bacterioplankton reveal years-long retention of Atlantic deep-ocean water by the Tropic Seamount

**DOI:** 10.1038/s41598-020-61417-0

**Published:** 2020-03-13

**Authors:** Greta Giljan, Nina A. Kamennaya, Andreas Otto, Dörte Becher, Andreas Ellrott, Volker Meyer, Bramley J. Murton, Bernhard M. Fuchs, Rudolf I. Amann, Mikhail V. Zubkov

**Affiliations:** 1 Max Planck Institute for Marine Microbiology, Bremen, Germany; 2 National Oceanography Centre, Southampton, UK; 3 Department of Microbial Proteomics, Institute for Microbiology, University Greifswald, Greifswald, Germany; 4 Present Address: School of Plant Sciences and Food Security, The George S. Wise Faculty of Life Sciences, Tel Aviv University, Tel Aviv, Israel; 5 Present Address: Scottish Association for Marine Science, Oban, Scotland UK

**Keywords:** Element cycles, Marine biology

## Abstract

Seamounts, often rising hundreds of metres above surrounding seafloor, obstruct the flow of deep-ocean water. While the retention of deep-water by seamounts is predicted from ocean circulation models, its empirical validation has been hampered by large scale and slow rate of the interaction. To overcome these limitations we use the growth of planktonic bacteria to assess the retention time of deep-ocean water by a seamount. The selected Tropic Seamount in the North-Eastern Atlantic is representative for the majority of isolated seamounts, which do not affect the surface ocean waters. We prove deep-water is retained by the seamount by measuring 2.4× higher bacterial concentrations in the seamount-associated or ‘sheath’-water than in deep-ocean water unaffected by seamounts. Genomic analyses of flow-sorted, dominant sheath-water bacteria confirm their planktonic origin, whilst proteomic analyses of the sheath-water bacteria, isotopically labelled *in situ*, indicate their slow growth. According to our radiotracer experiments, it takes the sheath-water bacterioplankton 1.5 years to double their concentration. Therefore, the seamount should retain the deep-ocean water for 1.8 years for the deep-ocean bacterioplankton to grow to the 2.4× higher concentration in the sheath-water. We propose that turbulent mixing of the seamount sheath-water stimulates bacterioplankton growth by increasing cell encounter rate with ambient dissolved organic molecules.

## Introduction

The 1,000-year-long global thermohaline circulation^1^ connects the bulk deep water of the modern World Ocean, irrespective of barriers erected by continents, islands and thousands of seamounts^2–4^. While continents and islands shape the circulation, seamounts affect this deep-water flow by creating enclosed circulation cells^5^, thereby reducing exchange between the so-called ‘sheath-water’ retained by seamounts and the surrounding deep water.

This does not, however, mean that the sheath-water is stagnant. The interaction of seamounts with deep water currents and waves (internal and tidal) causes complex sheath-water dynamics^6^, specified by the unique geometry of individual seamounts^5^. The complexity arises from interactions of parallel, rapid, turbulent mixing at centimetre-scales on seamount slopes^7,8^ and much slower flowing circulations (including Taylor columns) at the seamount-scale^9^. The complex sheath-water dynamics shapes seamount habitats for resident benthos and plankton^5,10,11^, causes erosion, controls sedimentation and affects ferromanganese crust formation on seamount slopes^12^.

A number of seamounts peak close to the ocean surface and mix nutrient-rich deep water with the nutrient-poor surface water enhancing local phytoplankton growth^13^ and causing a surface seamount effect^5,13,14^; retention of the produced organic matter in the seamount proximity raises productivity and enriches diversity of the entire seamount-associated ecosystem^9,10,15–19^. The majority of seamounts do not cause the surface seamount effect because their summits are hundreds or even thousands of meters below the sea surface. Because water retention at seamount-scales is challenging to measure directly, or to assess indirectly through combining observations with high-resolution hydrodynamic modelling^9,12,20^, the existence of the sheath-water remains a theoretical concept awaiting empirical proof. Measuring the duration of sheath-water retention would help determine the magnitude of interactions between individual seamounts and deep water currents.

Here, we propose a biological approach to test the sheath-water concept and to assess the sheath-water retention time. The most suitable proxy organisms are ubiquitous bacterioplankton, because they grow exclusively on organic molecules dissolved in the deep-ocean water and can be enumerated with high accuracy and precision by flow cytometry^21^. To ensure that sheath-water bacterioplankton, rather than suspended benthic bacteria, were analysed we compared bacterial abundance in samples collected from more than 5 m above the bottom to those in water samples carefully collected by a remotely operated vehicle (ROV) ~1 m above the bottom. Furthermore we assessed the composition of bacterioplankton for potential “contamination” with re-suspended benthic bacteria using high-throughput sequencing of flow sorted cells^22^. In the absence of established methods for reliably determining growth of deep-ocean bacterioplankton, we combined two different approaches. First, a custom-built, deep-water incubator was employed to identify those proteins the sheath-water bacterioplankton cells synthesise *in situ*. Second, we adapted the bioassay approach using amino acid radiotracers at close to ambient concentrations^23^ for ship-board incubations of deep-water samples.

We combined these techniques in a case study of bacterioplankton growth in the sheath-water of a typical, standalone seamount – Tropic Seamount located in the North-Eastern tropical Atlantic Ocean^24^. Tropic Seamount was selected because it is sufficiently (~3,000 m) tall to retain deep-ocean water yet its summit is deep enough below the ocean surface (~1,000 m) to cause no pronounced surface seamount effect, which would unnecessary complicate the already challenging assessment.

## Material and Methods

### Studied areas

**Figure 1:**
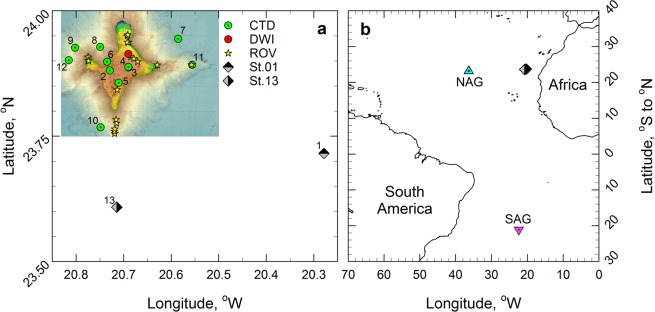
Locations of the sampling sites in the Atlantic Ocean. (**a)** The sampling sites at Tropic Seamount (St. 2–12) and in the seamount periphery (St. 1 and 13) (Supplementary Table S1): where water samples were collected using bottles mounted on the conductivity-temperature-depth (CTD) profiler at the seamount (green circles) and its periphery (grey-black diamonds); where the samples were incubated with ^13^C-lysine *in situ* using the deep-water incubator (DWI, the red circle); where water samples were collected using bottles mounted on the remotely operated vehicle (ROV, yellow stars). The cold colour gradient indicates the total relief and the warm colour gradient indicates the plateau topography on top of the seamount at higher resolution. (**b**) The sampling sites in the seamount-free middle North and South Atlantic subtropical gyres (NAG and SAG, respectively) relative to Tropic Seamount (Stations 1 and 13).

This study was conducted in the area of Tropic Seamount (23 °50′N, 20 °40′W) in the tropical North Atlantic on board the Royal Research Ship (RRS) *James Cook* (cruise number JC142) from 28^th^ October to 6^th^ December 2016 (Fig. 1). This standalone, compact (50 km wide), ferromanganese crust-covered guyot rises steeply from the 4,100 m deep abyssal plane to a 990 m flat summit plateau^12^. Water samples were collected with a sampling rosette of 20-litre Niskin bottles mounted on a conductivity-temperature-depth (CTD) profiler from the sea surface down to about 5 m above the seafloor (Stations 2–12) and within 1 meter above the seafloor by the ROV, called Isis (Fig. 1a, Supplementary Tables S1 and S2). The two peripheral, south-eastern and southern stations (Stations 1 and 13) served as the local reference of Tropic Seamount. The stations in the middle of the North and South Atlantic subtropical gyres (23 °8.4′N, 36 °21′W and 21 °6′S, 22 °23′W, respectively, Fig. 1b) served as external references. These stations were sampled during the Atlantic Meridional Transect on board the RRS *Discovery* (cruise number AMT17-D299) from 15^th^ October to 28^th^ November 2005.

### Bacterioplankton cell counting by flow cytometry and biomass estimation

Seawater samples were fixed with 2% (w/v) paraformaldehyde for 1 h at room temperature (RT). Bacterial cells were stained with SYBR Green I DNA dye^25^ and counted by flow cytometry (FACSort flow cytometer, Becton Dickinson) using the CellQuest software. Bacterioplankton concentrations were determined using 0.5 μm yellow-green micro-spheres (Fluoresbrite Microparticles, Polyscience) as an internal standard^26^. To convert bacterioplankton concentrations (N, cells l^−1^) into biomass (B) we used a mean cellular biomass (B_c_) of 11.5 fg carbon or protein cell^−1^ ^27^:

1 $${\rm{B}}\,[{\rm{g}}\,{\rm{C}}\,{\rm{or}}\,{\rm{protein}}\,{{\rm{l}}}^{-1}]={{\rm{B}}}_{{\rm{c}}}\times {\rm{N}}$$

under the assumption of equal cell carbon and protein contents^28^.

### Flow cytometric sorting of dominant bacterioplankton population

Deep-ocean bacterioplankton cells were concentrated from approximately 3 litre volumes directly from Niskin bottles using sterile 0.2 μm Sterivex filter units (Millipore, Watford). Concentrated samples were fixed with Lugol iodine solution and discoloured with thiosulfate solution^29^ before being stained with the DNA-specific Hoechst 33342 dye at a final concentration of 0.1 μg ml^−1^. Stained samples were analysed and target populations flow sorted with the custom-configured MoFlo XDP instrument (Beckman-Coulter, High Wycombe, UK) using the Summit 4.0 software as described previously^22^. Briefly, bacterial populations were visualised using a combination of the forward angle light scatter [FSC] of a UV diode laser (355 nm, 100 mW; JDSU, CY355-100, Thailand) and the dye fluorescence at 457 ± 25 nm. The flow cytometric plot was drawn using the FlowJo® v10 flow cytometric analysis software (Tree Star).

### Molecular identification of flow sorted bacterial populations

For taxonomic identification, ~2 × 10^3^ sorted cells from the main bacterial populations were added to 30 μl of Q5 High Fidelity Master Mix (New England BioLabs) complemented with primers and nuclease-free water (Ambion). The V3-V4 hyper-variable regions (490 bp) of 16S rRNA gene were PCR-amplified using S-D-Bact-0341-b-S-17 and S-D-Bact-0785-a-A-21 primers^30^ tailed with the Ion Torrent sequencing adapters. The forward primer also included the PGM barcode adapter (Ion Xpres Barcode Adapters 1–96 Kit, Thermo- Fisher Scientific). PCR products (~490 bp) were gel purified with NucleoSpin Gel and PCR Cleanup kit (Macherey-Nagel, Düren), pooled and used as template for emulsion PCR with the Ion Torrent One-Touch System (ThermoFisher Scientific) at a concentration of 26 pmol l^−1^. Sequencing of PCR products was done on an Ion Torrent PGM sequencer (ThermoFisher Scientific) using the Hi-Q sequencing chemistry. Sequences were quality trimmed for sequence length (>300 bp), controlled for homopolymers (<2%) and ambiguities (<2%) and separated by barcode using mothur^31^. Taxonomic affiliation was extracted by sequence comparison to the SSU rRNA SILVA database 119 using the SILVAngs pipeline^32^.

### The *in situ* incubation experiment for bacterioplankton proteomic analyses

**Figure 2:**
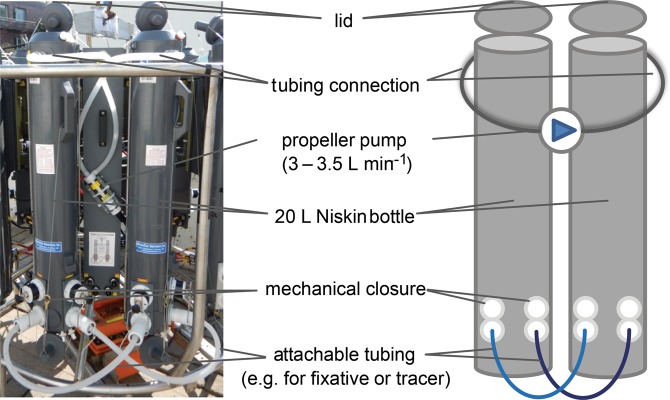
The deep-water *in situ* incubator design. The incubator is a tethered water sampling rosette of twelve 20 L Niskin bottles with lids arranged into six sets of paired bottles. A set (a photo to the left, a scheme to the right) consists of two bottles connected at the top by a tubing line with an in-line propeller pump. The two tubing lines at the bottom are deployed closed and disengaged from the bottles. The bottom tubing line can be simultaneously opened and attached to the paired bottles using the two mechanical closures. Before deployment each tubing line is filled with water solution contained fixative or tracer. The incubator can be either operated via a seabird deck unit or pre-programmed for autonomous operation at a target depth.

The custom-built deep-water *in situ* incubator (Fig. 2, depth rated to 6,000 m) was designed for *in situ* incubation experiments at depth to avoid decompression artefacts. For the bacterioplankton proteomic experiment the incubator was operated using the on deck control unit. The bottles were closed at 1,064 m and the tubing connectors were engaged to add ^13^C-Lysine tracer at 10 nmol l^−1^ final concentration. Pumps were switched on to mix the tracer with sampled water, closed in the paired bottles. After 20 hour incubation with the tracer *in situ* the incubator was recovered on board ship. Bacterioplankton were concentrated using 0.2 μm Sterivex filter units and prepared for sorting on board as described above. For targeted proteomic analysis, 2–5 × 10^6^ cells were flow sorted directly onto 0.2 µm filters and stored at −80 °C until further processing ashore.

### Proteomic analyses of bacterioplankton populations

Peptides were extracted from the sorted cells deposited on filters by repeated freeze-thawing to lyse the cells and subsequent tryptic protein digestions with sequencing grade modified Trypsin (Promega, Madison, Wisconsin). Cell debris was removed by centrifugations and peptides were concentrated and desalted on Millipore C18 ZipTip column (Merck Millipore, Darmstadt, Germany). Peptides were separated by EASY-nLCII (Thermo Fisher Scientific, Waltham, Massachussets) with self-packed analytical columns (100 μm × 20 cm) containing C18 RP material (Phenomenex, Aschaffenburg, Germany) and measured on an LTQ Orbitrap Elite (Thermo Fisher Scientific). Based on data dependent MS/MS mode, a Full Scan was done using the Orbitrap analyzer, followed by the analysis of the 10 most intense precursor ions using the LTQ analyzer. Singly charged ions were not taken into account for MS/MS analysis and lock mass option was enabled throughout all analyses. Data processing and protein identification based on this reference database was done with the Sorcerer 2 (SageN research) software. Taxonomic hits from 16S taq-sequencing of sorted populations were used to create a dataset specific protein reference database, containing 3049 entries, with publicly available genomic sequences from environmental samples and cultured representatives. Data analysis was done with the Scaffold 4 (version Scaffold_4.4.8, Proteome Software, Inc., Portland, Oregon) software. Identified proteins of the expressed proteome were manually classified into categories of orthologous groups based on general function categories. To convert leucine uptake rate into biomass production by bacterioplankton leucine content in measured peptides was calculated: on average leucine made up 6.86% of all amino acids by weight or 7.31 mol %.

### Microbial amino acid uptake rates assayed using radiotracer dilutions

The ambient bioavailable concentrations of amino acids, leucine and lysine, and microbial uptake rates of these amino acids were estimated using the radiotracer dilution bioassay^33,34^. Subsamples of 1.6 ml from the surface mixed layer (25 m) were incubated with either 0.1, 0.2, 0.4, 0.6, 0.8 and 1.0 nmol L-[3,5-^3^H] leucine (103 Ci mmol^−1^, Hartmann Analytics) or L-[3,5-^3^H] lysine (32 Ci mmol^−1^, Hartmann Analytics) at RT for 15, 30, 45 and 60 min. To increase sensitivity of measurements in deep waters (>900 m) 1 litre samples were amended with ^3^H-leucine or ^3^H-lysine to final concentrations 0.01, 0.025, 0.05, 0.075, 0.1 nmol l^−1^ and incubated at *in situ* temperature (Supplementary Fig. S1). Subsamples of 250 ml were withdrawn after approximately 6, 14, 22 and 30 h. Subsamples were fixed with 2% PFA at RT for 1 h. The particulate material was collected onto 0.2 μm pore size polycarbonate filters (Nucleopore, Whatman) and rinsed twice with deionised water. Filters were placed in plastic 8 ml scintillation vials. Vials were filled with 5 ml of Gold Star scintillation cocktail (Meridian Biotechnologies, Tadworth). Radioactivity retained on the filters was radio-assayed using a liquid scintillation counter (Tri-Carb 3100, Perkin Elmer). Leucine and lysine uptake rates of bacterioplankton were determined using a linear regression model (Supplementary Fig. S1).

### Computation of bacterioplankton production using the leucine uptake rates

To convert the leucine uptake rates into bacterioplankton production (P)^35^ under the same assumption of equal cell carbon and protein contents^28^ the leucine uptake rates (LUR, mol leucine l^−1^ d^−1^, multiplying the hourly rates by 24 h) were multiplied by the molecular weight of leucine (131.2 g) and divided by the fraction of leucine (7.31 mol%/100) in bacterial proteins:

2 $${\rm{P}}\,[{\rm{g}}\,{\rm{C}}\,{\rm{or}}\,{\rm{protein}}\,{{\rm{d}}}^{-1}]={\rm{LUR}}\times 131.2\times (100/7.31)$$

We assume bacterioplankton acquire all leucine exogenously from the leucine-depleted (e.g. 0.05 nmol leucine l^−1^ concentrations, Supplementary Fig. S1) sheath-waters. The 7.31 mol% value derived from our mass spectrometric analysis of bacterioplankton proteins (see above) is in good agreement with the leucine 7.3 ± 1.91 mol% value determined by high performance liquid chromatography analysis^35^.

### Assessment of the sheath-water retention time using the bacterioplankton growth

The sheath-water retention time equals the time required for bacterioplankton to grow from the concentrations in the seamount periphery (N_perif_) or in the deep-ocean (N_deep_) to the concentrations in the seamount sheath-water (N_sheath_). Therefore to assess the retention time we need to determine two values: (**i**) the above concentration difference (∆N_sp_ = N_sheath_ − N_perif_, ∆N_pd_ = N_perif_ − N_deep_ and ∆N_sd_ = N_sheath_ − N_deep_) and (**ii**) the doubling or generation time (DT) of bacterioplankton in the sheath-water. Because bacterioplankton growth in the sheath-water is restricted by availability of organic nutrients (e.g. Supplementary Fig. S1) we compared the restricted (by both nutrients and mortality), linear growth model with the generally used, unrestricted, exponential model of bacterial growth^28^ to assess the doubling time extremes.

Linear doubling time (LDT = 1/*k*, where *k* is the specific growth rate) of bacterioplankton in days (or years) equals bacterial biomass B [g C or protein l^−1^] divided by bacterial production P [g C or protein l^−1^ d^−1^ or year^−1^]:

3 $${\rm{LDT}}={\rm{B}}/{\rm{P}}$$

Exponential doubling time (EDT or *g*) of bacterioplankton in days (or years) equals linear doubling time LDT multiplied by natural logarithm of 2:

4 $${\rm{EDT}}={\rm{LDT}}\times \,\mathrm{ln}\,2$$

In brief the Eq. 4 derived from the following equation: ln(2B_0_) − ln(B_0_) = EDT/LDT where B_0_ is biomass at time zero and 2B_0_ equated to B_0_ biomass doubling. The latter equation can be simplified as ln(2B_0_/B_0_) = ln2 = EDT/LDT. See the chapter five of the Ingraham, *et al*.^28^ for details.

The seamount sheath-water retention time required for bacterioplankton to grow linearly (LRT) or exponentially (ERT) from e.g. N_deep_ to N_sheath_ was calculated according to the following equations:

5 $${\rm{LRT}}={\rm{LDT}}\times {\Delta {\rm{N}}}_{{\rm{sd}}}/2$$

6 $${\rm{ERT}}={\rm{LDT}}\times \,\mathrm{ln}({\Delta {\rm{N}}}_{{\rm{sd}}})$$

## Results and Discussion

### Concentration and phylogenetic characterisation of the sheath-water bacterioplankton

**Figure 3:**
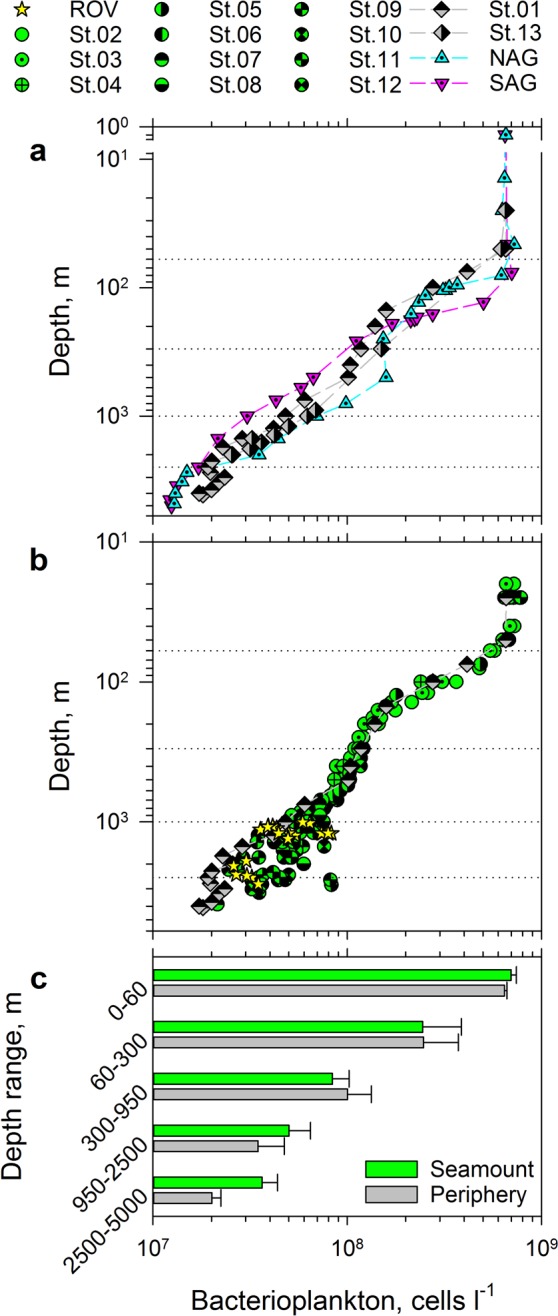
Bacterioplankton distributions in the water column at Tropic Seamount. (**a**) Comparison of bacterioplankton concentrations between the water column in Tropic Seamount periphery (St. 1 and 13) and the North and South Atlantic gyres (NAG and SAG, respectively). (**b**) Comparison of bacterioplankton concentrations between samples collected within 1 m of the seamount surface by a remote operated vehicle (ROV), at Tropic Seamount (St. 2–12) and in its periphery (St. 1). Dotted horizontal lines (**a**,**b**) indicate the five water layers defined. (**c**) Comparison of mean bacterioplankton concentrations in the five water layers between Tropic Seamount and its periphery. Error bars indicate single standard deviations of mean values. The results of the corresponding statistical analyses are presented in Supplementary Table S4. The corresponding sampling locations are shown in Fig. 1.

An assessment of bacterioplankton enrichment in the sheath-water of Tropic Seamount requires adequate reference sites (Fig. 1). Because the extent of the sheath-water was unknown, we used a nested approach of double referencing: (i) vertical profiles of bacterioplankton concentrations in the seamount-free areas of the North and South Atlantic gyres served as an external reference (Figs. 1b and 3a), (ii) vertical profiles of bacterioplankton concentrations at the seamount periphery (47 km south-east from the seamount summit centre to Station 1 and 24 km south to Station 13) served as a local reference (Figs. 1a and 3a,b). Bacterial concentrations within 1 m (ROV) and ≥5 m (CTD) above the seamount surface were compared to assess dispersal of re-suspended benthic bacteria in the sheath-water (Figs. 1a and 3b).

As would be expected, vertical distribution of bacterioplankton at 300–2,000 m depths in the seamount periphery was more similar to the distribution in the geographically closer North Atlantic subtropical gyre than in the geographically distant South Atlantic subtropical gyre (Fig. 3a). Below 2,500 m bacterioplankton concentrations in the two Atlantic gyres are statistically undistinguishable (Supplementary Table S3), whilst being 1.5× lower than the bacterioplankton concentrations in the seamount periphery (Fig. 3a, Supplementary Table S3). This indicates that the seamount affects bacterioplankton concentration even at the local reference sites: the seamount sheath-water extends beyond 20–40 km from the seamount summit (Fig. 1a). This bias of the local reference is taken into account in the latter analyses.

To assist comparisons, we divided the water column into five layers (Fig. 3), of which the two lowest are punctuated by Tropic Seamount. Bacterioplankton concentrations in the top three layers were similar above the seamount and in its periphery (Supplementary Table S4) confirming the laminar flow of water above the seamount and the minimal seamount effect on overlying waters. Indeed, a Taylor column (that can cause this effect) above Tropic Seamount has been found to be weak^12^. In the bottom two layers, bacterioplankton concentrations were significantly higher (1.5 and 1.8 times, respectively) at the seamount than in the seamount periphery (Fig. 3a,c), indicating the existence of the bacterioplankton-enriched sheath-water.

**Figure 4:**
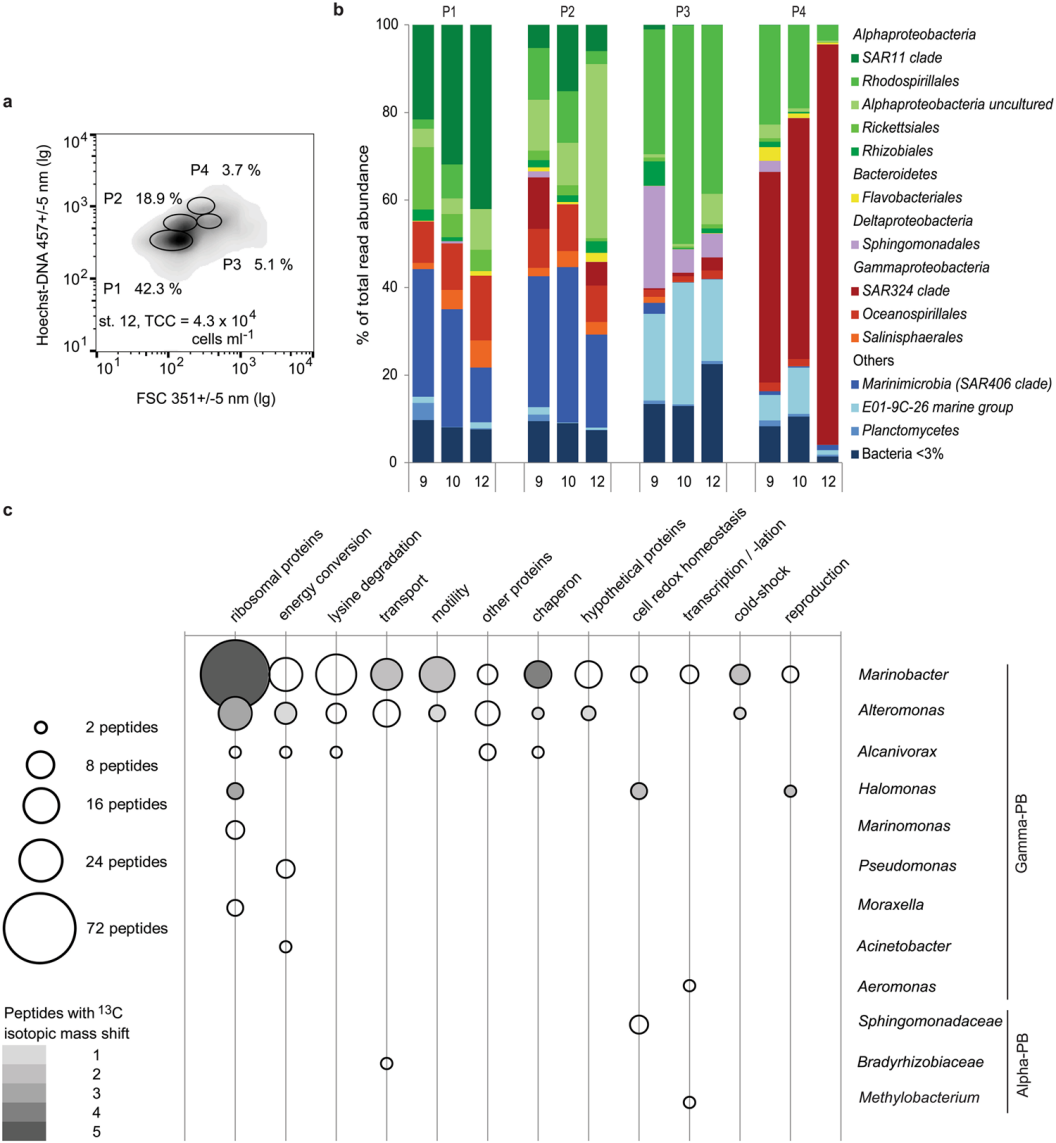
Taxonomic and proteomic characterisation of flow sorted bacterioplankton populations sampled from the seamount sheath-water. (**a)** Characteristic flow cytometric signatures of Hoechst–DNA stained bacterioplankton, sampled at St. 12. Ellipses on the density plot indicate the four main bacterioplankton populations (P1, P2, P3 and P4 with corresponding relative abundances shown as percentages), from which cells were flow sorted for taxonomic and proteomic analyses. The total cell concentration (TCC) is presented for reference. (**b**) Comparison of average (n = 2) relative taxonomic composition (% of read abundance) of the four main cytometric populations of bacterioplankton (BPL) sampled at the stations 9 (2,653 m), 10 (3,215 m) and 12 (3,030 m). P1 is dominated by the Rhodospirillales, Sphingomonadales and E01-9C-26 group. P2 is reproducibly dominated by the SAR11 and SAR406 groups. P3 is dominated by more diverse Alphaproteobacteria in addition to the SAR406 group. P4 is dominated by the SAR324 group. (**c**) Functional versus taxonomic classification of the peptides synthesised by cells from P1, focusing on the representative alpha- and gamma- proteobacteria (PB). The limited peptide data reveal the presence of bifunctional aconitate hydratase, 3-hydroxyacyl-CoA dehydrogenase, succinyl-CoA synthetase, isocitrate dehydrogenase, aspartate aminotransferase, aconitate hydratase and malate dehydrogenase enzymes and proteins responsible for core cellular functions such as protein expression, energy conservation, membrane transport or DNA folding.

To test whether the observed higher bacterial concentrations at the seamount are caused by re-suspended benthic bacteria we compared bacterioplankton concentrations in samples collected ≥5 m (CTD) and ~1 m (ROV) above the slope (Fig. 3a). Because the two datasets were statistically indistinguishable (Supplementary Table S5) the uncertainty remained: the benthic bacteria could be evenly suspended farther than 5 m above the seabed or their presence in bacterioplankton could be insignificant. To resolve the uncertainty we decided to characterise bacterioplankton taxonomically. However, by collecting bacterioplankton cells on filters, we would also collect particles suspended from the seafloor, marine snow particles and other particulate materials, which could bias taxonomic composition of truly bacterioplankton community. To avoid this bias we assessed bacterial diversity of the four main flow sorted bacterioplankton populations (Fig. 4a). To test for homogeneity of the sheath-water bacterioplankton we compared the bacterial diversity within the four populations at three locations above the seamount (2,653, 3,030 and 3,215 m depth). We focused on Bacteria as molecularly and ecologically better characterised group (compared to Archaea or protists) in the deep-ocean. Furthermore, compared to eukaryotes basic metabolic rates and growth of prokaryotes could be directly assessed by the uptake rates of amino acids.

Variation in composition and relative abundance of bacterial taxa in each of the four flow cytometric population was low between the three locations (Fig. 4b). The results of taxonomic analyses confirmed the dominance of planktonic bacteria and negligible content of potentially benthic bacteria (Fig. 4b, Supplementary Table S6) independent of sampling depth and location. Specifically, there was no indication that obligate benthic species^36–38^ (e.g. JTB255) were present among the sheath-water bacteria, whilst the dominant identified taxa (e.g. *SAR11*, *Rhodospirillales, SAR324* and *SAR406*) are commonly found in deep-ocean bacterioplankton^39,40^ (Supplementary Table S6). Hence, the analysed sheath-water bacteria were indeed planktonic.

### Proteins synthesized *in situ* by dominant cells of the sheath-water bacterioplankton

To ascertain whether the sheath-water bacterioplankton are actively growing, we analysed their protein synthesis (basic metabolism) *in situ*. We incubated isolated water samples in the deep-water incubator (Fig. 2) with ^13^C_6_-lysine label to identify the newly synthesized proteins. Because analyses of metaproteomic libraries remain challenging^41^, we used flow sorting that guaranteed us targeted proteomic analyses of the two most abundant populations of sheath-water bacterioplankton (the 1^st^ and 2^nd^ population, Fig. 4a) enabling direct metabolic and growth assessment of the bacterioplankton majority.

Low rates of ^3^H-lysine uptake by the sheath-water bacterioplankton at the ambient lysine concentration (see Supplementary Fig. S1) guided us to add 10 nmol l^−1^ of ^13^C_6_-lysine to overcome potential detection limitation. Even at this artificially high (>2,000 times higher than ambient) concentration we could only detect labelled peptides in cells flow sorted from the 1^st^ population, and the labelled peptides had an isotopic mass shift of merely 5–30%. Approximately ten percent of detected peptides belonged to Gammaproteobacteria related to *Alteromonas sp., Marinomonas sp*. and *Halomonas sp*. (Fig. 4c). With *Halomonas sp*. being among the most abundant (Supplementary Table S6), all the three taxa were represented in the molecular data of the 1^st^ population, validating our proteomic analyses (Fig. 4b,c). The newly synthesised proteins were involved in cell maintenance (ribosomal proteins, chaperones, proteins involved in energy conversion, cold-shock proteins) and active substrate uptake (membrane transporter proteins, flagellar proteins).

Active synthesis of proteins involved in transcription and translation, rather than in replication of nucleic acids (Fig. 4c), indicates preferential cellular maintenance rather than cell division. An isotopic mass shift, present in all detected peptides of cold shock proteins, indicates their constant regeneration. This is likely to reflect the mechanism of cell adaptation to cold (2–7 °C) deep-ocean conditions. Lysine addition predictably induced sheath-water bacteria to synthesise proteins involved in amino acid transport and utilization. Irrespective the artificial nature of this induction we could conclude that multiple taxa of sheath-water bacterioplankton are metabolically active and responsive to nutrient pulses (Fig. 4c), suggesting that availability of organic nutrients could restrict their growth.

### Amino acid acquisition and growth of the sheath-water bacterioplankton

To reduce artificial stimulation of bacterial metabolic activity^23^ in samples incubated on board ship, we traced amino acid (leucine and lysine) uptake at concentrations close to ambient. We found that in the surface waters bacterioplankton uptake of leucine was 3 times faster than lysine uptake, whilst in the sheath-water this difference rose to >100 (Supplementary Fig. S1). Owing to its higher specific activity, leucine is a more sensitive tracer than lysine for assessing bacterial growth particularly at lower rates measured in the sheath-water. Consequently, we base our assessments of bacterial metabolic activity and growth on the results of experiments with the leucine tracer.

**Figure 5:**
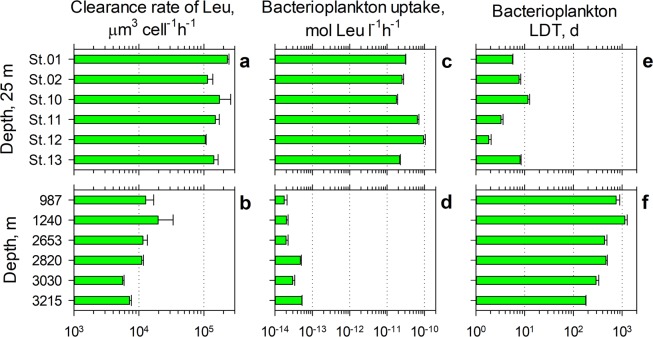
Bacterioplankton growth in the surface and sheath- waters of Tropic Seamount. Comparison of cellular clearance rates (**a**,**b**), bacterioplankton uptake rates of leucine (Leu) (**c**,**d**) and bacterioplankton linear doubling time (LDT) (**e**,**f**) between the surface mixed layer (25 m) above Tropic Seamount (Stations 2, 10–12) and in periphery (Stations 1 and 13) of the seamount (**a**,**c**,**e**) and in the sheath-water (**b**,**d**,**f**).

The amino acid uptake rate of a bacterial cell indicates its rate of protein synthesis and overall cellular metabolic activity. Therefore leucine clearance rates (the volume of water cleared of dissolved, bioavailable leucine by a cell in an hour) allows direct comparison of cell-specific metabolic activity of bacterioplankton living in the surface (Fig. 5a, Supplementary Table S7) and sheath- waters (Fig. 5b). The leucine clearance rates demonstrate that the metabolic activity of the sheath-water bacterioplankton cell is relatively high and, on average, 7.4% of the metabolic activity of the surface water bacterioplankton cell. However, because the sheath-water bacterioplankton standing stock is only 7.5% of the bacterioplankton standing stock in the surface waters (Fig. 3c, Supplementary Table S1), the standing stock-specific uptake of leucine in the sheath-water (Fig. 5d) is merely 0.074% of the uptake in the surface waters (Fig. 5c, Supplementary Table S7), underlining the challenge of measuring bacterioplankton growth in the deep-ocean waters.

According to the measurements of leucine uptake (42 ± 30 pmol l^−1^ h^−1^, mean ± SD, Fig. 5c), the surface waters above Tropic Seamount are about three times more productive than the surface waters of the oligotrophic Atlantic gyres (13 ± 3 pmol l^−1^ h^−1^) and of comparable productivity to the tropical surface waters (40 ± 14 pmol l^−1^ h^−1^)^42,43^ unaffected by upwelling. This independently supports our earlier conclusion that Tropic Seamount has virtually no effect on bacterioplankton in the overlying waters. The leucine uptake rates were converted into bacterial production and the linear doubling time according to the Eqs. 1–3 using corresponding concentrations (Fig. 3c, Supplementary Table S1) and leucine uptake rates of bacterioplankton (Fig. 5c,d, Supplementary Table 7).

The calculated mean doubling time (Fig. 5e, LDT = 6.4 ± 3.6 days, EDT = 4.4 ± 2.5 days) of bacterioplankton in the surface waters above Tropic Seamount is comparable to bacterioplankton doubling in the open ocean (11 ± 15 d)^44^, in the temperate North Atlantic (7.1–12.5 d)^45^ or in the oligotrophic ocean (10–20 d)^46^. Compared to the surface waters bacterioplankton in the sheath-water of Tropic Seamount double ninety times slower, once every 543 ± 348 days (Fig. 5f). The mean LDT = 1.5 ± 1.0 years or EDT = 1.0 ± 0.66 years is within the broad range of 0.1–30 years^47,48^ estimates for deep-ocean bacterioplankton. Comparatively high cellular metabolic activity of the sheath-water bacterioplankton (5a, b) in conjunction with their slow growth (Fig. 5f) is in agreement with the proteomics results of effective intracellular recycling of main proteins rather than active bacterial reproduction (Fig. 4c).

Based on the determined LDT of 1.5 years and concentration difference (1.5× and 1.8× in the bottom two layers, Fig. 3c; on average ∆N_sp_ = 1.6×), it would take bacterioplankton in the seamount periphery 0.7 = 1.5 × ln(1.6) years and 1.2 = 1.5 × 1.6/2 years to grow to the concentration of bacterioplankton in the sheath-water using the exponential (Eq. 6) or linear (Eq. 5) model, respectively. The exponential model gives an estimate for the fastest, unrestricted bacterioplankton growth, whilst the more realistic linear model accounts for such restrictions^28^ and therefore should better approximate bacterioplankton growth in the seamount sheath-water.

Bacterioplankton concentration in the seamount periphery is, however, still 1.5× higher than in the deep-ocean waters unaffected by seamounts, ∆N_pd_ = 1.5× (Fig. 3a). Therefore, it would take the deep-ocean bacterioplankton between ERT = 1.3 = 1.5 × ln(1.6 × 1.5) years and LRT = 1.8 = 1.5 × (1.6 × 1.5)/2 years to grow to reach the concentration of bacterioplankton in the sheath-water. To enable bacterioplankton to grow from the deep-ocean to the seamount sheath-water concentrations Tropic Seamount should retain the deep-water in its sheath for 1.3–1.8 years. This provides the original, experimentally-derived estimate of the years-long interaction between a representative seamount and the deep-ocean water flow.

Even the maximal 1.8-year retention is a short time compared with the 1,000 year global thermohaline circulation^1^. However, considering that the North Atlantic deep-water components of northern origin spread throughout the western North Atlantic within 25–30 years^49^, 1.8-year seamount sheath-water retention time is significant. Higher concentrations of bacterioplankton cells in the sheath-water (Fig. 3b,c) could explain what controls growth of deep-ocean bacterioplankton. Because there is no extra source of dissolved organic molecules in the sheath-water of Tropic Seamount, bacterioplankton consume bio-available, dissolved molecules. The only difference between the sheath- and surrounding deep- water is the complex sheath-water dynamics that includes intensive turbulent mixing of the former^7,8^ compared to laminar-flowing deep-water in seamount-free areas. Therefore, it would be turbulent water mixing that ultimately controls bacterioplankton growth in the deep ocean: growth of the deep-ocean bacterioplankton is limited by the bioavailability of organic molecules in cell vicinity and turbulent mixing alleviates that limitation. Furthermore, the longer the sheath-water is retained by a seamount, the more organic molecules in the retained water are consumed by resident bacterioplankton. The consequences of such interactions and their impact on the seamount surface is worth exploring in follow-up studies.

## Supplementary information

Supplementary Information.

## Data Availability

The Ion Torrent-generated libraries of bacterial 16S rRNA gene sequences were archived at the European Nucleotice Archive (ENA) (https://www.ebi.ac.uk/ena) of The European Bioinformatics Institute (EMBL-EBI) with the International Nucleotide Sequence Database Collaboration (INSDC) accession number PRJEB35653. Flow Cytometry datasets are archived at the FlowRepository database (https://flowrepository.org), ID: FR-FCM-Z2D9. The mass spectrometry proteomics data have been deposited to the ProteomeXchange Consortium (http://www.ebi.ac.uk/pride) via the Proteomics Identification Database (PRIDE) partner repository with the dataset identifier PXD016702.
