# Supplementary material for: Bacterioplankton reveal years-long retention of Atlantic deep-ocean water by the Tropic Seamount
Source: Sci Rep. 2020 Mar 13;10:4715. doi: 10.1038/s41598-020-61417-0 (PMC7069937; doi:10.1038/s41598-020-61417-0)
Supplement: Supplementary file 1 — Supplementary Information. [file 41598_2020_61417_MOESM1_ESM.pdf]

Supplementary Materials for

Bacterioplankton reveal years-long retention of Atlantic deep-ocean water by the Tropic Seamount

G Giljan, NA Kamennaya, A Otto, D Becher, A Ellrott, V Meyer, BJ Murton, BM Fuchs, RI Amann & MV Zubkov*

Supplementary Figure S1

Supplementary Tables S1-S7

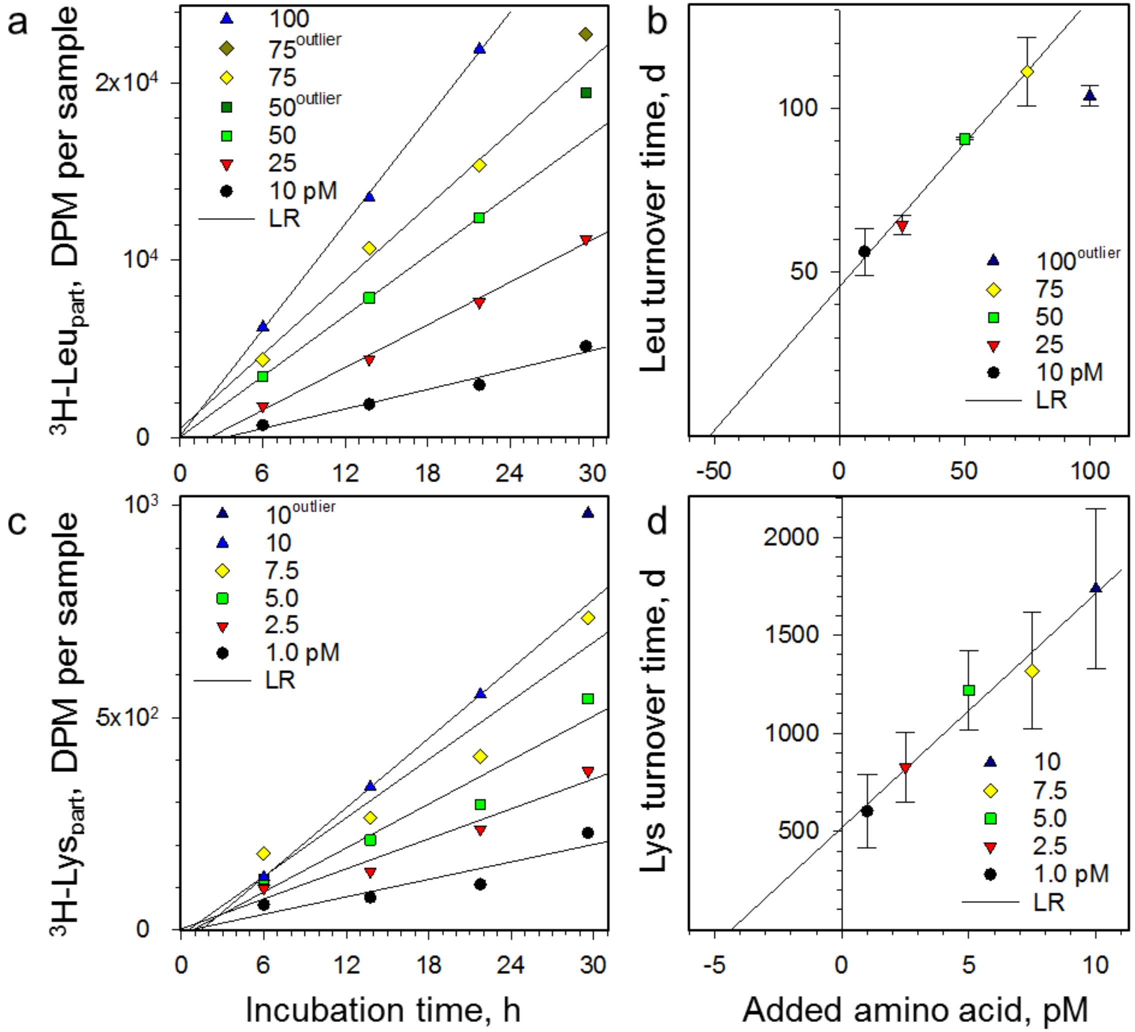

Supplementary Figure S1.

Bioassay estimation of bacterioplankton uptake rates of leucine (Leu) and lysine (Lys) at ambient concentrations in a sheath-water sample, collected at Tropic Seamount (Station 12) from a depth of 2820 m.

a, c. Time series at different **(a)** Leu and **(c)** Lys concentrations with linear regression lines (LR); bacterioplankton uptake was estimated in a series, in which different amounts of ^{3}H-Leu **(a)** or ^{3}H-Lys **(c)** were added to samples.

b, **d**. Relationships between added Leu **(b)** and Lys **(d)** concentrations and turnover times of dissolved Leu or Lys pools; the LR slope estimates the uptake rate: 1.14 ±0.07 pmol Leu $l^{-1}d^{-1}$ **(b)** and 0.0096 ±0.0011 pmol Lys l^{-1} d^{-1} **(d)**; y-axis intercept of LR is an estimate of the turnover time: 45.5 ±2.7 d for Leu and 565 ±90 d for Lys, at ambient Leu or Lys concentrations: 51.9 ±4.5 pM and 5.4 ±1.1 pM, respectively, which is x-axis intercept **(b, d)**.

Considering the total bacterioplankton concentration of 82.8 $\times 10^6$ cells l^{-1} the corresponding cellular uptake rates are 345 ±22 molecules Leu cell^{-1} h^{-1} **(b)** and 2.9 ±0.3 molecules Lys cell^{-1} h^{-1} **(d)**.

Supplementary Table S1.

CTD sampling station and bacterioplankton concentrations determined using flow cytometry, as shown on Fig. 3a,b.

Station number	Date	Lat. [N]	Long. [W]	Depth [m]	Cells/ml	Depth [m]	Cells/ml	Depth [m]	Cells/ml	Depth [m]	Cells/ml
1	31Oct2016	23° 42.930	20° 16.725	25	658000	300	118000	1500	28800	3000	23400
				50	654000	400	103000	1750	22800	3250	21900
				75	414000	500	101000	2000	25700	3750	20000
				100	276000	750	60300	2250	20100	4000	17300
				150	158000	1000	48000	2500	19200	4100	18100
				200	139000	1250	41700	2750	19700		
2	01Nov2016	23° 53.209	20° 41.365	20	722000	140	215000	350	104000	700	78000
				40	724000	160	177000	400	96900	800	71900
				60	573000	180	149000	450	98100	850	57400
				80	479000	200	145000	500	103000	900	56200
				100	364000	250	121000	550	94500	950	50700
				120	260000	300	115000	600	92300	987	50000
3	03Nov2016	23° 52.840	20° 43.733	20	658000	160	143000	500	94600	775	76600
				40	688000	180	135000	600	89200	800	62800
				60	544000	200	123000	650	82900	900	51600
				100	309000	250	115000	700	72900	1000	48600
				120	244000	300	109000	725	73300	1085	71800
				140	169000	400	87700	750	74600		
4	07Nov2016	23° 53.269	20° 41.399	25	657000	300	114000	650	82000	991	72100
				50	631000	400	95400	700	81100		
				100	239000	500	85800	800	66500		
				200	136000	600	86900	900	62100		
5	08Nov2016	23° 51.375	20° 42.629	25	680000	200	136000	600	91400	800	76100
				50	680000	300	121000	650	84800	900	58400
				75	487000	350	117000	675	86100	1006	53300
				100	275000	400	108000	700	88700		
				125	179000	450	103000	725	77800		
				150	158000	550	101000	750	77900		
6	09Nov2016	23° 53.892	20° 44.080	700	74500	800	66700	1299	45000		
				750	73500	900	59000				

7	10Nov2016	23° 56.635	20° 35.125	25	718000	1250	34700	3889	21400		
				750	72000	2000	25700				
8	18Nov2016	23° 55.671	20° 44.959	900	72300	1200	69300	1600	46700	2000	59700
				1000	75400	1400	42100	1800	50200		
9	21Nov2016	23° 55.569	20° 48.138	25	687000	1500	59100	2200	27400	2653	32300
				1000	59200	1600	55100	2400	36800		
				1200	57300	1800	35000	2500	35200		
				1400	34300	2000	27300	2610	44100		
10	26Nov2016	23° 46.050	20° 44.906	25	773000	1800	47200	2800	36100		
				1500	76000	2400	50000	3000	35000		
				1600	54600	2600	47900	3215	35200		
11	28Nov2016	23° 53.528	20° 33.354	25	784000	1400	53000	2500	45000		
				1000	64200	1800	51700	2600	81900		
				1200	66900	2300	41500	2820	82800		
12	30Nov2016	23° 54.080	20° 48.920	25	646000	1000	62300	2200	24600		
				400	117000	1400	46500	2800	36400		
				800	62900	1800	29100	3030	32300		
13	02Dec2016	23° 36.500	20° 42.858	25	652000	900	68700	1400	42100	1800	31600
				50	619000	1000	62200	1500	32406	2000	25400
				300	150000	1200	49800	1600	36100		

Supplementary Table S2.

ROV water sampling and bacterioplankton concentration determined using flow cytometry, as shown on Fig. 3b.

Date	Lat. [N]	Long. [W]	Depth [m]	Cells/ml
14Nov2016	23° 45.295	20° 43.126	2501	32200
	23° 45.575	20° 43.189	2422	30400
	23° 45.866	20° 43.116	2382	26800
	23° 46.552	20° 42.881	2066	26000
	23° 46.992	20° 42.946	1898	30200
15Nov2016	23° 54.477	20° 41.107	1022	64000
	23° 54.282	20° 40.721	1019	57800
	23° 53.902	20° 40.230	1006	58900
16Nov2016	23° 56.239	20° 41.395	1136	35800
	23° 56.230	20° 41.554	1112	43300
	23° 56.990	20° 41.561	1076	41300
	23° 56.990	20° 41.561	1076	39200
	23° 57.125	20° 41.470	1087	39000
18Nov2016	23° 50.540	20° 42.806	1029	74400
	23° 50.540	20° 42.806	1029	79800
	23° 50.540	20° 42.806	1029	82600
	23° 50.540	20° 42.806	1029	73600
	23° 50.540	20° 42.806	1029	79700
21Nov2016	23° 54.457	20° 46.483	1253	53800
	23° 54.038	20° 46.462	1209	50000
	23° 54.010	20° 46.433	1208	44300
28Nov2016	23° 53.548	20° 33.416	2786	34200
	23° 53.548	20° 33.416	2786	34800
	23° 53.548	20° 33.416	2786	34900
29Nov2016	23° 53.515	20° 37.755	1318	49700
	23° 53.515	20° 37.755	1318	49800
	23° 53.515	20° 37.755	1318	49500

Supplementary Table S3.

Results of the two-tailed *t*-test analyses testing the similarity in bacterioplankton concentrations between the deep layers of the water column in Tropic Seamount periphery and the seamount-free middle North and South Atlantic subtropical gyres, as shown on Fig. 3a.

Seamount-free	Periphery	*Student's* t-test, *t*	Normality test Shapiro-Wilks, *P*	Equal variance test Brown-Forsythe, *P*	Degrees of freedom	*P*	Power of performance with *P*=0.050
950-2,500 (6)	950-2,500 (14)	0.233	passed, 0.163	passed, 0.477	18	0.818	0.05
2,500-4,000 (7)	2,500-4,000 (6)	-7.189	passed, 0.841	passed, 0.168	11	0.0000178	1.0

Bacterioplankton concentrations in the seamount periphery were statistically undistinguishable and 1.51 times higher than in the seamount-free deep ocean waters in the layers 950-2500 m and 2500-4000 m, respectively.

Supplementary Table S4.

Results of the two-tailed *t*-test analyses testing the similarity in bacterioplankton concentrations in five layers of the water column between Tropic Seamount and its periphery, as shown on Fig. 3b,c.

Seamount	Periphery	*Student's* t-test, *t*	Normality test Shapiro-Wilks, *P*	Equal variance test Brown-Forsythe, *P*	Degrees of freedom	*P*	Power of performance with *P*=0.050
0-60 m (13)	0-60 m (4)	2.069	passed, 0.515	passed, 0.095	15	0.0563	0.49
60-300 m (24)	60-300 m(4)	*	failed, <0.05	passed, 0.49	26	0.718	
300-950 m (52)	300-950 m (6)	-1.9	passed, 0.865	passed, 0.097	56	0.0625	0.463
950-2,500 (38)	950-2,500 (14)	3.58	passed, 0.571	passed, 0.586	50	0.000775	0.94
2,500-4,000 (11)	2,500-4,000 (6)	5.309	passed, 0.076	passed, 0.132	15	0.0000875	0.999

* Mann-Whitney Rank Sum Test.

Bacterioplankton concentrations in the top three water layers were similar between the seamount and its periphery. Bacterioplankton concentrations in the seamount sheath-water were 1.45 and 1.82 times higher than in the seamount periphery in the layers 950-2500 m and 2500-4000 m, respectively.

Supplementary Table S5.

Results of the two-tailed *t*-test analyses testing the similarity in bacterioplankton concentrations between samples collected using the ROV versus CTD near the top and along the slopes of Tropic Seamount, as shown on Fig. 3b.

ROV	CTD	*Student's* t-test, *t*	Normality test Shapiro-Wilks, *P*	Equal variance test Brown-Forsythe, *P*	Degrees of freedom	*P*	Power of performance with *P*=0.050
950-1,300 (13)	950-1,300 (5)	1.254	passed, 0.139	passed, 0.6	16	0.228	0.218
2,000-3,000 (6)	2,000-3,000 (3)	1.591	passed, 0.924	passed, 0.583	7	0.156	0.28

Bacterioplankton concentrations in samples collected using the ROV and CTD were statistically undistinguishable in both layers.

Supplementary Table S6.

Isolation source overview of the most abundant bacterioplankton taxa (>0.5 % read abundance). Comparison of all isolation sources based on the SSU rRNA SILVA database 119 with a focus on the pelagic and benthic marine habitat. Highlighted in bold is the marine habitat with most reference sequences per taxon. The relative abundance in % of the given taxa in all analysed populations is presented.

Taxonomic affiliation (>0.5% read abundance)	Isolation source				Relative read abundance [%]											
					Station 9				Station 10				Station 12			
	Pelagic	Benthic	Other / unknown	Total	P1	P2	P3	P4	P1	P2	P3	P4	P1	P2	P3	P4
Proteobacteria Alphaproteobacteria Rhizobiales Bradyrhizobiaceae Afipia	**1**	**1**	142	144	0.0	0.0	0.0	0.0	0.2	0.5	0.4	0.3	0.0	0.0	0.0	0.0
Proteobacteria Alphaproteobacteria Rhizobiales Bradyrhizobiaceae Bradyrhizobium	**14**	11	1442	1467	0.2	0.0	0.0	0.1	1.3	1.9	2.1	1.0	0.0	0.0	0.0	0.0
Proteobacteria Alphaproteobacteria Rhizobiales Methylobacteriaceae Methylobacterium	**10**	**10**	895	915	0.0	0.0	0.0	0.0	0.0	0.0	0.0	0.0	3.8	3.8	0.0	0.0
Proteobacteria Alphaproteobacteria Rhizobiales Phyllobacteriaceae Hoeflea	**25**	4	47	76	0.0	0.5	0.0	0.0	0.6	0.5	0.7	0.8	0.0	0.0	0.0	0.0
Proteobacteria Alphaproteobacteria Rhizobiales Phyllobacteriaceae Mesorhizobium	**18**	4	758	780	0.3	3.9	0.0	0.0	2.3	0.2	0.1	0.1	0.1	0.0	0.0	0.0
Proteobacteria Alphaproteobacteria Rhizobiales PS1 clade	**3**	0	0	3	0.0	0.0	0.0	0.0	1.2	1.2	0.9	1.4	0.0	0.0	0.0	0.0
Proteobacteria Alphaproteobacteria Rhizobiales Rhodobiaceae Parvibaculum	**3**	2	36	41	2.9	0.1	6.8	0.5	2.1	2.7	1.8	3.5	0.0	0.0	0.0	0.0
Proteobacteria Alphaproteobacteria Rhizobiales Rhodobiaceae uncultured	**7**	5	80	92	0.0	0.0	0.7	0.0	0.5	0.5	0.8	0.5	0.0	0.0	0.0	0.0
Proteobacteria Alphaproteobacteria Rhodospirillales AT-s3-44	**48**	8	18	74	0.0	0.0	0.0	0.2	0.3	1.0	1.3	2.0	1.7	0.0	0.4	0.0
Proteobacteria Alphaproteobacteria Rhodospirillales Rhodospirillaceae AEGEAN-169 marine group	**62**	0	17	79	0.0	0.0	0.0	0.0	0.1	0.1	0.1	0.1	0.3	0.9	0.0	0.0
Proteobacteria Alphaproteobacteria	**28**	14	22	64	0.0	0.0	0.0	0.2	0.1	0.3	0.7	0.8	8.8	0.0	0.0	0.0

Rhodospirillales Rhodospirillaceae Magnetospira																
Proteobacteria Alphaproteobacteria Rhodospirillales Rhodospirillaceae Magnetovibrio	**8**	7	7	22	0.0	0.0	0.0	0.0	1.3	1.1	1.1	1.1	0.0	0.0	0.0	0.0
Proteobacteria Alphaproteobacteria Rhodospirillales Rhodospirillaceae Thalassobaculum	**1**	0	14	15	0.0	25.4	0.0	0.0	8.2	0.4	0.1	0.0	0.0	0.0	0.0	0.0
Proteobacteria Alphaproteobacteria Rhodospirillales Rhodospirillaceae uncultured	**703**	118	796	1617	3.7	1.3	36.2	88.0	28.4	31.7	33.5	20.6	6.9	0.9	4.0	0.0
Proteobacteria Alphaproteobacteria Rickettsiales	**0**	**0**	5	5	0.0	0.0	0.0	0.0	0.7	0.7	0.7	0.7	0.3	0.3	0.0	0.6
Proteobacteria Alphaproteobacteria Rickettsiales LWSR-14	**2**	0	40	42	0.0	0.0	1.9	0.0	4.0	4.2	3.7	4.0	0.0	0.0	0.0	0.0
Proteobacteria Alphaproteobacteria Rickettsiales S25-593	**76**	0	15	91	0.0	0.0	1.7	0.0	0.9	1.1	1.0	1.0	3.3	0.3	0.0	6.2
Proteobacteria Alphaproteobacteria Rickettsiales TK34	1	**14**	26	41	0.0	0.0	0.0	0.0	0.3	0.5	0.6	0.6	0.5	0.0	0.0	0.0
Proteobacteria Alphaproteobacteria SAR11 clade	**433**	3	20	456	0.0	0.0	0.4	0.0	0.7	0.5	0.5	0.5	2.9	0.2	0.0	5.6
Proteobacteria Alphaproteobacteria SAR11 clade Deep 1	**404**	0	17	421	0.0	0.0	3.1	0.0	5.3	5.6	4.5	3.6	8.0	1.5	0.0	14.5
Proteobacteria Alphaproteobacteria SAR11 clade Surface 1	**2662**	2	176	2840	0.0	0.0	4.3	0.0	3.1	3.3	3.5	3.5	9.4	3.5	0.0	24.6
Proteobacteria Alphaproteobacteria SAR11 clade Surface 4	**96**	0	10	106	0.0	0.0	0.8	0.0	0.0	0.0	0.0	0.0	0.0	0.0	0.0	0.0
Proteobacteria Alphaproteobacteria Sphingomonadales Erythrobacteraceae Altererythrobacter	**17**	13	293	323	16.0	0.6	0.0	0.7	3.8	3.7	0.5	0.3	0.0	0.0	0.0	0.0
Proteobacteria Alphaproteobacteria Sphingomonadales Erythrobacteraceae Erythrobacter	**135**	38	784	957	0.5	0.4	0.0	0.4	1.9	5.4	5.5	5.2	0.0	0.0	0.0	0.0
Proteobacteria Alphaproteobacteria Sphingomonadales Erythrobacteraceae uncultured	**19**	10	188	217	0.0	0.0	0.0	0.0	0.0	0.5	1.0	1.0	0.0	0.0	0.0	0.0

Proteobacteria Alphaproteobacteria Sphingomonadales Sphingomonadaceae Parasphingopyxis	**2**	1	10	13	62.3	4.5	5.9	3.5	12.0	13.1	4.0	3.6	2.2	0.0	0.0	0.0
Proteobacteria Alphaproteobacteria Sphingomonadales Sphingomonadaceae Sphingomonas	**62**	21	2769	2852	1.4	0.9	0.0	0.6	0.7	0.7	0.0	0.0	0.0	0.0	0.0	0.0
Proteobacteria Alphaproteobacteria Sphingomonadales Sphingomonadaceae Sphingorhabdus	**24**	5	56	85	0.2	0.2	0.0	0.7	0.5	0.4	0.5	0.7	0.0	0.0	0.0	0.0
Proteobacteria Alphaproteobacteria uncultured	**9667**	203	3951	13821	0.5	0.0	0.0	0.2	2.8	3.7	3.4	6.8	10.8	5.1	29.2	0.4
Bacteroidetes Flavobacteriia Flavobacteriales Cryomorphaceae Owenweeksia	**149**	10	128	287	0.0	0.0	0.0	0.0	0.0	0.0	0.8	2.4	0.2	0.0	0.0	0.0
Bacteroidetes Flavobacteriia Flavobacteriales NS9 marine group	**173**	1	128	302	0.0	0.0	1.8	0.0	0.0	0.0	0.0	0.0	0.4	0.5	0.0	0.6
Proteobacteria Deltaproteobacteria SAR324 clade(Marine group B)	**208**	38	102	348	0.4	0.0	0.0	0.8	0.3	0.3	6.2	18.4	23.7	2.2	89.9	0.1
Proteobacteria Gammaproteobacteria Alteromonadales Alteromonadaceae Alteromonas	**401**	146	1252	1799	0.0	0.0	0.0	0.0	0.0	0.0	0.0	1.0	0.0	0.0	0.0	0.0
Proteobacteria Gammaproteobacteria Alteromonadales Alteromonadaceae Marinobacter	**197**	181	616	994	0.0	0.5	0.0	0.0	0.1	0.2	0.3	0.3	1.0	0.1	0.0	0.0
Proteobacteria Gammaproteobacteria E01-9C-26 marine group	**88**	22	267	377	0.4	0.8	0.0	0.2	1.1	6.2	7.0	8.1	5.6	0.5	1.4	1.0
Proteobacteria Gammaproteobacteria Oceanospirillales Alcanivoracaceae Alcanivorax	**107**	22	173	302	2.8	0.7	0.2	0.2	1.4	1.2	0.3	0.3	1.1	0.0	0.0	0.0
Proteobacteria Gammaproteobacteria Oceanospirillales Halomonadaceae Halomonas	**118**	198	1381	1697	0.0	0.4	0.0	0.0	0.0	0.0	0.9	0.9	0.0	0.0	0.0	0.0
Proteobacteria Gammaproteobacteria Oceanospirillales JL-ETNP-Y6	**23**	0	2	25	0.0	0.0	1.0	0.0	1.2	0.8	1.1	0.9	2.6	2.2	0.0	7.2
Proteobacteria Gammaproteobacteria Oceanospirillales Oceanospirillaceae	**102**	32	117	251	0.0	0.0	0.3	0.2	0.0	0.0	0.3	0.6	0.1	0.4	0.0	0.0

Pseudohongiella																
Proteobacteria Gammaproteobacteria Oceanospirillales SAR86 clade	**641**	0	72	713	0.0	0.0	0.4	0.0	0.6	0.6	0.5	0.4	0.5	0.2	0.0	1.2
Proteobacteria Gammaproteobacteria Oceanospirillales ZD0405	**86**	0	8	94	0.0	0.0	2.5	0.3	1.6	1.6	1.7	1.7	1.9	2.3	0.0	3.0
Proteobacteria Gammaproteobacteria Salinisphaerales Salinisphaeraceae Oceanococcus	**3**	0	1	4	2.7	1.8	0.0	0.3	0.9	0.8	0.3	0.3	0.0	0.0	0.0	0.0
Proteobacteria Gammaproteobacteria Salinisphaerales Salinisphaeraceae ZD0417 marine group	**43**	0	6	49	0.0	0.0	2.0	0.0	0.4	0.8	0.9	1.1	2.7	2.2	0.0	5.9
Marinimicrobia (SAR406 clade)	**313**	26	122	461	0.0	0.1	18.7	0.0	7.9	8.9	12.8	13.0	8.9	19.6	1.0	14.8
Planctomycetes Phycisphaerae CCM11a	5	**25**	63	93	0.0	1.9	0.0	0.0	0.0	0.0	0.0	0.0	0.0	0.0	0.0	0.0
Planctomycetes Phycisphaerae Phycisphaerales Phycisphaeraceae JL-ETNP-F27	**22**	0	6	28	0.0	0.0	0.6	0.0	0.0	1.2	1.1	1.1	0.1	0.0	0.1	0.0
Planctomycetes Phycisphaerae Phycisphaerales Phycisphaeraceae SM1A02	**9**	8	210	227	0.0	0.0	0.0	0.0	0.7	0.0	0.0	0.0	0.0	0.0	0.0	0.0
Planctomycetes Planctomycetacia Planctomycetales Planctomycetaceae Rhodopirellula	**51**	63	244	358	0.0	0.0	0.0	0.0	0.0	0.0	1.9	1.1	0.3	0.0	0.3	0.0
Planctomycetes Planctomycetacia Planctomycetales Planctomycetaceae uncultured	**176**	161	2333	2670	0.0	0.0	0.0	0.0	0.4	0.4	0.2	0.2	0.1	0.0	0.2	0.0

Supplementary Table S7.

Comparison of bacterioplankton metabolic rates in the surface mixed layer (25 m) at Tropic Seamount and in the seamount sheath-water (≥987 m): clearance rates of ambient leucine by a mean bacterioplankton cell, uptake rate of leucine by total bacterioplankton, bacterioplankton linear doubling time (LDT). The values are presented as mean ± standard error.

Depth [m]	Leu [μm^3 $cell^{-1}$ h^{-1}]	$\times 10^{-11}$ mol Leu l^{-1} h^{-1}	LDT [d]
25	230000 ±12600	3.11 ±0.04	5.65 ±0.08
25	114000 ±23000	2.52 ±0.23	7.66 ±0.71
25	173000 ±85000	1.77 ±0.15	11.7 ±0.97
25	150000 ±22500	6.43 ±0.69	3.25 ±0.35
25	105000 ±3470	9.42 ±1.12	1.83 ±0.22
25	142000 ±23400	2.16 ±0.11	8.06 ±0.41
Depth [m]	Leu [μm^3 $cell^{-1}$ h^{-1}]	$\times 10^{-14}$ mol Leu l^{-1} h^{-1}	LDT [d]
987	12700 ±4110	1.77 ±0.32	754 ±136
1240	19800 ±13900	2.01 ±0.23	1135 ±131
2653	11500 ±1960	1.98 ±0.25	436 ±54.6
2820	11100 ±664	4.75 ±0.3	466 ±29.4
3030	5610 ±274	2.95 ±0.41	292 ±40.8
3215	7140 ±505	5.24 ±0.08	179 ±2.65
